# Correction: Rimoldi et al. The Replacement of Fish Meal with Poultry By-Product Meal and Insect Exuviae: Effects on Growth Performance, Gut Health and Microbiota of the European Seabass, *Dicentrarchus labrax*. *Microorganisms* 2024, *12*, 744

**DOI:** 10.3390/microorganisms12081524

**Published:** 2024-07-25

**Authors:** Simona Rimoldi, Ambra Rita Di Rosa, Rosangela Armone, Biagina Chiofalo, Imam Hasan, Marco Saroglia, Violeta Kalemi, Genciana Terova

**Affiliations:** 1 Department of Biotechnology and Life Sciences, University of Insubria, 21100 Varese, Italy; simona.rimoldi@uninsubria.it (S.R.); ihasan@uninsubria.it (I.H.); marco.saroglia@gmail.com (M.S.); vkalemi@uninsubria.it (V.K.); 2 Department of Veterinary Sciences, University of Messina, 98168 Messina, Italy; ambra.dirosa@unime.it (A.R.D.R.); rosangela.armone@studenti.unime.it (R.A.); biagina.chiofalo@unime.it (B.C.)

In the original publication [1], there was a typographic error in the legend for Figure 2. The corrected legend appears below:

Figure 2. Standard haematoxylin–eosin (H&E) histochemical analysis of liver from representative images of fish fed control, PM20, and PM25 diets. We obtained 5 µm cross-sections from the paraffin-embedded liver of CTRL (panels (**A**,**B**)), PM20 (panels (**C**,**D**)), and PM25 (panels (**E**,**F**)) fish. Scale bar = 100 µm.

In the original publication [1], there was a mistake in Table 4. The data for the proximal and distal intestines were identical. Whilst the distal intestine data are correct, the proximal gut data were updated. Also, the term “submucosal layer thickness” was replaced with “muscular thickness”.

The corrected Table 4 appears below. 

The authors state that the scientific conclusions are unaffected. This correction was approved by the Academic Editor. The original publication has also been updated. 

## Figures and Tables

**Table 4:** Intestinal morphometric parameters of seabass fed experimental diets.

	CTRL	PM20	PM25	*p*-Value
Proximal intestine				
ViH (μm)	1446.26 ± 61.90	1434.69 ± 44.01	1445.89 ± 54.71	0.985
ViW (μm)	259.24 ± 11.65	244.82 ± 20.69	223.73 ± 22.12	0.4071
LPW (μm)	54.19 ± 3.21	51.74 ± 2.76	51.93 ± 4.38	0.862
MT (μm)	161.22 ± 6.35	169.39 ± 8.14	177.17 ± 8.49	0.29
Distal intestine				
ViH (μm)	1008.78 ± 21.50	1064.90 ± 37.14	1035.87 ± 31.11	0.435
ViW (μm)	78.35 ± 2.57	83.18 ± 3.05	84.79 ± 3.42	0.300
LPW (μm)	28.44 ± 1.00	29.64 ± 1.42	27.3 ± 1.78	0.585
MT (μm)	352.65 ± 14.93	365.94 ± 18.56	332.02 ± 9.22	0.266

Notes: villus height (ViH), villus width (ViW), lamina propria width (LPW), and muscular thickness (MT). Values are expressed as mean ± standard error.

## References

[1] Rimoldi S., Di Rosa A.R., Armone R., Chiofalo B., Hasan I., Saroglia M., Kalemi V., Terova G. (2024). The Replacement of Fish Meal with Poultry By-Product Meal and Insect Exuviae: Effects on Growth Performance, Gut Health and Microbiota of the European Seabass, *Dicentrarchus labrax*. Microorganisms.

