# Supplementary material for: The Replacement of Fish Meal with Poultry By-Product Meal and Insect Exuviae: Effects on Growth Performance, Gut Health and Microbiota of the European Seabass, Dicentrarchus labrax
Source: Microorganisms. 2024 Apr 6;12(4):744. doi: 10.3390/microorganisms12040744 (PMC11052083; doi:10.3390/microorganisms12040744)
Supplement: Supplementary file 1 [file microorganisms-12-00744-s001.zip › microorganisms-2924489-supplementary/Table S1.pdf]

Table S1. The list of bacterial genera that differ between experimental feeds A (control) and B.

PHYLUM	CLASS	ORDER	FAMILY	GENUS	A (%)	SD (%)	B (%)	SD (%)	p-value
Actinobacteriota	Actinobacteria	Micrococcales	AKAU3644	AKAU3644	0.01	0.01	0.64	0.02	0.0000
Actinobacteriota	Actinobacteria	Micrococcales	Dermabacteraceae	Brachybacterium	0.16	0.06	1.44	0.07	0.0001
Actinobacteriota	Actinobacteria	Actinomycetales	Actinomycetaceae	Actinomyces	0.19	0.11	1.20	0.10	0.0006
Actinobacteriota	Actinobacteria	Micrococcales	Brevibacteriaceae	Brevibacterium	0.28	0.13	1.70	0.06	0.0012
Actinobacteriota	Actinobacteria	Micrococcales	Microbacteriaceae	Leucobacter	0.06	0.02	0.43	0.04	0.0019
Actinobacteriota	Actinobacteria	Micrococcales	Micrococcaceae	Enteractinococcus	0.05	0.02	0.19	0.01	0.0023
Actinobacteriota	Actinobacteria	Corynebacteriales	Dietziaceae	Dietzia	0.06	0.01	0.66	0.07	0.0070
Actinobacteriota	Actinobacteria	Corynebacteriales	Corynebacteriaceae	Corynebacterium	1.62	0.74	3.72	0.19	0.0497
Bacteroidota	Bacteroidia	Bacteroidales	Dysgonomonadaceae	Dysgonomonadaceae	0.03	0.01	0.36	0.02	0.0000
Bacteroidota	Bacteroidia	Sphingobacteriales	Sphingobacteriaceae	Sphingobacterium	0.30	0.13	2.62	0.10	0.0001
Bacteroidota	Bacteroidia	Flavobacteriales	Flavobacteriaceae	Myroides	1.06	0.23	3.89	0.13	0.0004
Bacteroidota	Bacteroidia	Bacteroidales	Dysgonomonadaceae	Dysgonomonas	0.17	0.12	1.33	0.07	0.0009
Bacteroidota	Bacteroidia	Sphingobacteriales	Sphingobacteriaceae	Pedobacter	0.02	0.02	0.13	0.02	0.0034
Bacteroidota	Bacteroidia	Flavobacteriales	Flavobacteriaceae	Ulvibacter	0.02	0.01	0.38	0.05	0.0059
Bacteroidota	Bacteroidia	Bacteroidales	Bacteroidaceae	Bacteroides	0.04	0.02	0.51	0.07	0.0066
Desulfobacterota	Desulfovibrionia	Desulfovibrionales	Desulfovibrionaceae	Desulfovibrio	0.00	0.01	0.14	0.01	0.0002
Firmicutes	Clostridia	Peptostreptococcales-Tissierellales	Sedimentibacteraceae	Sedimentibacter	0.01	0.01	0.11	0.00	0.0007
Firmicutes	Bacilli	Bacillales	Bacillaceae	Pseudogracilibacillus	0.70	0.23	2.72	0.18	0.0008
Firmicutes	Bacilli	Lactobacillales	Enterococcaceae	Enterococcus	0.10	0.05	0.63	0.06	0.0009
Firmicutes	Bacilli	Staphylococcales	Staphylococcaceae	Staphylococcus	1.43	0.44	5.38	0.23	0.0014
Firmicutes	Bacilli	RsaHf231	RsaHf231	RsaHf231	0.01	0.01	0.16	0.02	0.0020
Firmicutes	Bacilli	Bacillales	Bacillaceae	Bacillus	1.15	0.12	2.09	0.16	0.0032
Firmicutes	Bacilli	Erysipelotrichales	Erysipelotrichaceae	Erysipelothrix	0.41	0.32	2.20	0.21	0.0043
Firmicutes	Bacilli	Bacillales	Bacillaceae	Amphibacillus	0.08	0.04	0.31	0.02	0.0062
Firmicutes	Clostridia	Peptostreptococcales-Tissierellales	Peptostreptococcaceae	Clostridioides	0.07	0.04	0.25	0.01	0.0153
Firmicutes	Clostridia	Lachnospirales	Lachnospiraceae	Lachnoclostridium	0.02	0.02	0.11	0.03	0.0273
Firmicutes	Clostridia	Oscillospirales	Ruminococcaceae	Incertae_Sedis	0.01	0.01	0.08	0.02	0.0300
Proteobacteria	Gammaproteobacteria	Enterobacterales	Morganellaceae	Providencia	1.14	0.62	12.29	0.56	0.0001

Proteobacteria	Gammaproteobacteria	Pseudomonadales	Pseudomonadaceae	Oblitimonas	0.19	0.12	1.32	0.14	0.0010
Proteobacteria	Gammaproteobacteria	Cardiobacteriales	Wohlfahrtiimonadaceae	Ignatzschineria	0.83	0.38	5.12	0.54	0.0013
Proteobacteria	Gammaproteobacteria	Enterobacterales	Yersiniaceae	Serratia	0.09	0.06	0.64	0.03	0.0017
Proteobacteria	Gammaproteobacteria	Burkholderiales	Alcaligenaceae	Paenalcaligenes	0.24	0.11	1.28	0.15	0.0019
Proteobacteria	Gammaproteobacteria	Aeromonadales	Aeromonadaceae	Aeromonas	0.15	0.09	1.97	0.20	0.0022
Proteobacteria	Gammaproteobacteria	Burkholderiales	Alcaligenaceae	Alcaligenes	0.26	0.02	1.47	0.12	0.0035
Proteobacteria	Gammaproteobacteria	Enterobacterales	Morganellaceae	Proteus	1.49	0.83	7.20	0.24	0.0068
Proteobacteria	Gammaproteobacteria	Enterobacterales	Morganellaceae	Morganella	0.12	0.07	1.99	0.33	0.0122
Proteobacteria	Alphaproteobacteria	Rhizobiales	Rhizobiaceae	Paenochrobactrum	0.08	0.05	0.41	0.06	0.0044
