# Supplementary material for: The Replacement of Fish Meal with Poultry By-Product Meal and Insect Exuviae: Effects on Growth Performance, Gut Health and Microbiota of the European Seabass, Dicentrarchus labrax
Source: Microorganisms. 2024 Apr 6;12(4):744. doi: 10.3390/microorganisms12040744 (PMC11052083; doi:10.3390/microorganisms12040744)
Supplement: Supplementary file 1 [file microorganisms-12-00744-s001.zip › microorganisms-2924489-supplementary/Table S2.pdf]

Table S2. The list of bacterial genera that differ between experimental feeds A (control) and C.

PHYLUM	CLASS	ORDER	FAMILY	GENUS	A (%)	SD (%)	C (%)	SD (%)	p-value
Actinobacteriota	Actinobacteria	Actinomycetales	Actinomycetaceae	Actinomyces	0.19	0.11	3.01	0.22	0.0006
Bacteroidota	Bacteroidia	Bacteroidales	Bacteroidaceae	Bacteroides	0.04	0.02	1.27	0.05	0.0001
Bacteroidota	Bacteroidia	Bacteroidales	Dysgonomonadaceae	Dysgonomonadaceae	0.03	0.01	1.93	0.06	0.0002
Bacteroidota	Bacteroidia	Bacteroidales	Dysgonomonadaceae	Dysgonomonas	0.17	0.12	2.90	0.44	0.0090
Bacteroidota	Bacteroidia	Flavobacteriales	Flavobacteriaceae	Myroides	1.06	0.23	0.10	0.04	0.0244
Firmicutes	Clostridia	Oscillospirales	Ruminococcaceae	Incertae_Sedis	0.01	0.01	0.23	0.01	0.0000
Firmicutes	Bacilli	Bacillales	Planococcaceae	Sporosarcina	0.41	0.17	2.54	0.21	0.0004
Firmicutes	Clostridia	Lachnospirales	Lachnospiraceae	Lachnoclostridium	0.02	0.02	0.17	0.02	0.0019
Firmicutes	Clostridia	Peptostreptococcales-Tissierellales	Sedimentibacteraceae	Sedimentibacter	0.01	0.01	0.11	0.01	0.0040
Firmicutes	Bacilli	Bacillales	Bacillaceae	Bacillus	1.15	0.12	21.97	1.98	0.0044
Firmicutes	Bacilli	Bacillales	Bacillaceae	Ureibacillus	0.23	0.10	12.00	1.37	0.0065
Firmicutes	Negativicutes	Acidaminococcales	Acidaminococcaceae	Phascolarctobacterium	0.01	0.01	0.14	0.04	0.0303
Firmicutes	Clostridia	Clostridiales	Clostridiaceae	Clostridium_sensu_stricto_18	0.10	0.03	0.02	0.02	0.0416
Firmicutes	Bacilli	RsaHf231	RsaHf231	RsaHf231	0.01	0.01	0.06	0.02	0.0443
Firmicutes	Bacilli	Staphylococcales	Staphylococcaceae	Staphylococcus	1.43	0.44	0.16	0.12	0.0460
Proteobacteria	Gammaproteobacteria	Burkholderiales	Alcaligenaceae	Alcaligenes	0.26	0.02	0.05	0.01	0.0027
