# Supplementary material for: The Replacement of Fish Meal with Poultry By-Product Meal and Insect Exuviae: Effects on Growth Performance, Gut Health and Microbiota of the European Seabass, Dicentrarchus labrax
Source: Microorganisms. 2024 Apr 6;12(4):744. doi: 10.3390/microorganisms12040744 (PMC11052083; doi:10.3390/microorganisms12040744)
Supplement: Supplementary file 1 [file microorganisms-12-00744-s001.zip › microorganisms-2924489-supplementary/Table S3.pdf]

Table S3. The list of bacterial genera that differ between experimental feeds B and C.

PHYLUM	CLASS	ORDER	FAMILY	GENUS	B (%)	SD (%)	C (%)	SD (%)	p-value
Actinobacteriota	Actinobacteria	Micrococcales	Brevibacteriaceae	Brevibacterium	1.70	0.06	0.07	0.03	4.41E-05
Actinobacteriota	Actinobacteria	Corynebacteriales	Corynebacteriaceae	Corynebacterium	3.72	0.19	0.46	0.17	5.54E-05
Actinobacteriota	Actinobacteria	Micrococcales	Dermabacteraceae	Brachybacterium	1.44	0.07	0.08	0.03	0.0003
Actinobacteriota	Actinobacteria	Micrococcales	Micrococcaceae	Enteractinococcus	0.19	0.01	0.01	0.01	0.0004
Actinobacteriota	Actinobacteria	Micrococcales	AKAU3644	AKAU3644	0.64	0.02	0.00	0.00	0.0004
Actinobacteriota	Actinobacteria	Micrococcales	Microbacteriaceae	Leucobacter	0.43	0.04	0.10	0.03	0.0015
Actinobacteriota	Actinobacteria	Actinomycetales	Actinomycetaceae	Actinomyces	1.20	0.10	3.01	0.22	0.0025
Actinobacteriota	Actinobacteria	Corynebacteriales	Dietziaceae	Dietzia	0.66	0.07	0.02	0.03	0.0032
Bacteroidota	Bacteroidia	Flavobacteriales	Crocinitomicaceae	Fluviicola	0.16	0.01	0.00	0.00	2.09E-05
Bacteroidota	Bacteroidia	Sphingobacteriales	Sphingobacteriaceae	Sphingobacterium	2.62	0.10	0.09	0.04	0.0001
Bacteroidota	Bacteroidia	Bacteroidales	Dysgonomonadaceae	Dysgonomonadaceae	0.36	0.02	1.93	0.06	0.0002
Bacteroidota	Bacteroidia	Flavobacteriales	Flavobacteriaceae	Myroides	3.89	0.13	0.10	0.04	0.0003
Bacteroidota	Bacteroidia	Bacteroidales	Bacteroidaceae	Bacteroides	0.51	0.07	1.27	0.05	0.0005
Bacteroidota	Bacteroidia	Flavobacteriales	Flavobacteriaceae	Flavobacterium	0.17	0.02	0.03	0.02	0.0021
Bacteroidota	Bacteroidia	Sphingobacteriales	Sphingobacteriaceae	Pedobacter	0.13	0.02	0.01	0.01	0.0041
Bacteroidota	Bacteroidia	Flavobacteriales	Flavobacteriaceae	Ulvibacter	0.38	0.05	0.09	0.06	0.0055
Bacteroidota	Bacteroidia	Bacteroidales	Dysgonomonadaceae	Dysgonomonas	1.33	0.07	2.90	0.44	0.0343
Firmicutes	Clostridia	Peptostreptococcales-Tissierellales	Peptostreptococcaceae	Clostridioides	0.25	0.01	0.01	0.01	0.0001
Firmicutes	Bacilli	Staphylococcales	Staphylococcaceae	Staphylococcus	5.38	0.23	0.16	0.12	0.0001
Firmicutes	Bacilli	Bacillales	Bacillaceae	Pseudogracilibacillus	2.72	0.18	0.85	0.12	0.0005
Firmicutes	Bacilli	Bacillales	Bacillaceae	Amphibacillus	0.31	0.02	0.05	0.01	0.0005
Firmicutes	Bacilli	Bacillales	Bacillaceae	Virgibacillus	0.23	0.01	0.14	0.01	0.0008
Firmicutes	Bacilli	Bacillales	Planococcaceae	Sporosarcina	0.74	0.11	2.54	0.21	0.0015
Firmicutes	Clostridia	Oscillospirales	Ruminococcaceae	Incertae_Sedis	0.08	0.02	0.23	0.01	0.0031
Firmicutes	Clostridia	Clostridiales	Clostridiaceae	Clostridium_sensu_stricto_7	0.00	0.00	0.04	0.00	0.0033
Firmicutes	Bacilli	Bacillales	Bacillaceae	Geobacillus	0.04	0.01	0.10	0.01	0.0038
Firmicutes	Bacilli	Lactobacillales	Enterococcaceae	Enterococcus	0.63	0.06	0.14	0.02	0.0038
Firmicutes	Bacilli	Bacillales	Bacillaceae	Bacillus	2.09	0.16	21.97	1.98	0.0047

Firmicutes	Bacilli	Erysipelotrichales	Erysipelotrichaceae	Erysipelothrix	2.20	0.21	0.40	0.05	0.0047
Firmicutes	Bacilli	Bacillales	Planococcaceae	Savagea	2.62	0.27	0.16	0.04	0.0052
Firmicutes	Bacilli	Lactobacillales	Streptococcaceae	Streptococcus	0.30	0.03	0.72	0.07	0.0057
Firmicutes	Bacilli	RsaHf231	RsaHf231	RsaHf231	0.16	0.02	0.06	0.02	0.0060
Firmicutes	Bacilli	Bacillales	Bacillaceae	Ureibacillus	0.19	0.05	12.00	1.37	0.0066
Firmicutes	Bacilli	Lactobacillales	Leuconostocaceae	Weissella	0.69	0.02	1.17	0.07	0.0070
Firmicutes	Bacilli	Lactobacillales	Carnobacteriaceae	Atopostipes	0.36	0.05	0.07	0.02	0.0077
Firmicutes	Bacilli	Bacillales	Bacillaceae	Cerasibacillus	0.14	0.01	0.07	0.01	0.0094
Firmicutes	Bacilli	Lactobacillales	Lactobacillaceae	Lactobacillus	16.56	0.76	35.81	3.29	0.0111
Firmicutes	Bacilli	Staphylococcales	Staphylococcaceae	Nosocomiicoccus	0.13	0.02	0.00	0.00	0.0159
Firmicutes	Bacilli	Bacillales	Bacillaceae	Oceanobacillus	0.24	0.04	0.09	0.01	0.0251
Fusobacteriota	Fusobacteriia	Fusobacteriales	Fusobacteriaceae	Cetobacterium	0.06	0.01	0.13	0.01	0.0042
Proteobacteria	Gammaproteobacteria	Enterobacterales	Yersiniaceae	Serratia	0.64	0.03	0.03	0.02	6.01E-05
Proteobacteria	Gammaproteobacteria	Enterobacterales	Morganellaceae	Proteus	7.20	0.24	0.07	0.02	0.0005
Proteobacteria	Gammaproteobacteria	Enterobacterales	Morganellaceae	Providencia	12.29	0.56	0.17	0.06	0.0009
Proteobacteria	Gammaproteobacteria	Pseudomonadales	Moraxellaceae	Acinetobacter	0.23	0.02	0.05	0.02	0.0010
Proteobacteria	Gammaproteobacteria	Burkholderiales	Alcaligenaceae	Alcaligenes	1.47	0.12	0.05	0.01	0.0032
Proteobacteria	Gammaproteobacteria	Burkholderiales	Alcaligenaceae	Paenalcaligenes	1.28	0.15	0.31	0.08	0.0038
Proteobacteria	Gammaproteobacteria	Aeromonadales	Aeromonadaceae	Aeromonas	1.97	0.20	0.06	0.02	0.0050
Proteobacteria	Gammaproteobacteria	Pseudomonadales	Pseudomonadaceae	Oblitimonas	1.32	0.14	0.04	0.01	0.0052
Proteobacteria	Gammaproteobacteria	Cardiobacteriales	Wohlfahrtiimonadaceae	Ignatzschineria	5.12	0.54	0.46	0.02	0.0066
Proteobacteria	Gammaproteobacteria	Enterobacterales	Morganellaceae	Morganella	1.99	0.33	0.08	0.04	0.0134
Proteobacteria	Alphaproteobacteria	Rhizobiales	Rhizobiaceae	Paenochrobactrum	0.41	0.06	0.07	0.01	0.0139
Proteobacteria	Gammaproteobacteria	Xanthomonadales	Xanthomonadaceae	Stenotrophomonas	0.18	0.04	0.05	0.02	0.0169
